# Supplementary material for: Bayesian longitudinal tensor response regression for modeling neuroplasticity
Source: Hum Brain Mapp. 2023 Nov 1;44(18):6326–48. doi: 10.1002/hbm.26509 (PMC10681668; doi:10.1002/hbm.26509)
Supplement: Supplementary file 1 — DATA S1: Supporting Information. [file HBM-44-6326-s001.docx]

Supplementary Materials for Bayesian longitudinal tensor response regression for modeling neuroplasticity

Suprateek Kundu, Department of Biostatistics, UT MD Anderson Cancer Center. Email: [skundu2@mdanderson.org](mailto:skundu2@mdanderson.org).

Alec Reinhardt,^[[1]](#footnote-1)^ Department of Biostatistics, UT MD Anderson Cancer Center. Email: [aereinhardt@mdanderson.org](mailto:aereinhardt@mdanderson.org).

Serena Song, Center for Visual and Neurocognitive Rehabilitation, Atlanta Veterans Affairs Medical Center. Email: [serena.eungy.song@emory.edu](mailto:serena.eungy.song@emory.edu)

Joo Han, Center for Visual and Neurocognitive Rehabilitation, Atlanta Veterans Affairs Medical Center. Email: [jhan42@student.gsu.edu](mailto:jhan42@student.gsu.edu)

M. Lawson Meadows, Center for Visual and Neurocognitive Rehabilitation, Atlanta Veterans Affairs Medical Center. Email: [lawsonmeadows1@gmail.com](mailto:lawsonmeadows1@gmail.com)

Bruce Crosson, Departments of Neurology and Imaging and Radiological Sciences, Emory University. Email: [brucecrosson2@gmail.com](mailto:brucecrosson2@gmail.com)

Venkatagiri Krishnamurthy, Division of Geriatrics and Gerontology, Emory University. Email: [venkatagiri@emory.edu](mailto:venkatagiri@emory.edu).

**Deviance Information Criteria for Tensor Rank Selection**

Given a choice of the tensor rank $R$, and the model parameters $\Psi^{k}=\{\mathcal{M}^{k},B_{i}^{k},\Gamma^{k},\Theta_{i}^{k},\mathcal{B}_{tm}^{k},\mathcal{D}_{s}^{k},\mathcal{C}_{q}^{k},\sigma_{\epsilon,ti}^{k}\}$ corresponding to the $k$th iteration, the deviance $D^{k}$ under Model (1) is expressed as

$$D^{k}=D\left( \{\mathcal{Y}_{ti}\left( v \right){\}}_{t\in\left[ 1,T \right],i\in\left[ 1,n \right],v\in\mathcal{V}_{i}} | \Psi^{k} \right)=-2\log P\left( \{\mathcal{Y}_{ti}\left( v \right)\} | \Psi^{k} \right)$$

$$=-2\log\prod_{t=1}^{T} \prod_{i=1}^{n} \prod_{v\in\mathcal{V}_{i}} P\left( \mathcal{R}_{ti}^{k}\left( v \right) | \sigma_{\epsilon,ti}^{k} \right)=\sum_{t=1}^{T} \sum_{i=1}^{n} \sum_{v\in\mathcal{V}_{i}} \left( \left( \frac{\mathcal{R}_{ti}^{k}\left( v \right)}{\sigma_{\epsilon,ti}^{k}} \right)^{2}+2\log\sigma_{\epsilon,ti}^{k}+\log\left( 2\pi\right) \right)$$

where $\mathcal{R}_{ti}^{k}\left( v \right)=\mathcal{Y}_{ti}\left( v \right)-\mathcal{M}^{k}\left( v \right)-B_{i}^{k}\left( v \right)-\left( \Gamma^{k}\left( v \right)+\Theta_{i}^{k}\left( v \right) \right)\mathcal{T}_{ti}-\sum_{m=1}^{M} \mathcal{B}_{tm}^{k}\left( v \right)c_{im}-\sum_{s=1}^{S} \mathcal{D}_{s}^{k}\left( v \right)x_{is}-\sum_{q=1}^{Q} \mathcal{C}_{q}^{k}z_{tiq}$ and $\mathcal{V}_{i}$ is the set of observed voxels $v$ for subject $i$ ($i=1,\ldots, n$). Denoting $\bar{D}$ as the mean value of $D^{k}$ across all post-burn-in MCMC iterations, $\bar{\Psi}$ as the posterior mean of model parameters across post-burn-in MCMC iterations, and $\{\mathcal{Y}_{ti}\}$ as the set of all tensor-valued outcomes $\mathcal{Y}_{ti}$ across all visits $t$, subjects $i$, and observed voxels $v$, the estimated effective number of parameters $pD$ is given by $pD=\bar{D}-D\left( \{\mathcal{Y}_{ti}\} | \bar{\Psi} \right)$. Moreover, the Deviance Information Criterion (DIC) is defined as $DIC=\bar{D}+pD$, where $\bar{D}$ penalizes poor model fits and $pD$ penalizes models with a large number of parameters resulting from large choice of rank $R$ (Shriner and Yi, 2009). The Bayesian model is fit repeatedly for different choices of the tensor rank, and the optimal rank is chosen corresponding to the model that returns the lowest DIC score.

**Posterior Computation Steps**

Suppose there are $n$ unique subjects, and that the $i$th subject has $T_{i}$ clinical visits for $i\in\left[ 1,n \right]$. For a given dimension $d\in\left[ 1,D \right]$ and tensor margin index $j\in\left[ 1,p_{d} \right]$, let $\mathcal{Y}_{ti,\cdot,dj}$ be the slice of $\mathcal{Y}_{ti,\cdot}$ obtained by fixing dimension $d$ at index $j$, and let $\mathcal{V}_{ti,dj}$ be the set of indices for observed voxels of this slice. Denote the squared Euclidean norm of a vector as $\left\| \cdot\right\|_{2}^{2}$. Then the sampling steps for the full MCMC algorithm for the l-BTRR method are as follows, where ranks $r\in\left[ 1,R \right]$ and dimensions $d\in\left[ 1,D \right]$ are looped through:

**Step 1**: Let $\mathcal{Y}_{ti,r}^{\mu}=\mathcal{Y}_{ti}-\hat{B}_{i}-\hat{\Gamma}\times\mathcal{T}_{ti}-\hat{\Theta}_{i}\times\mathcal{T}_{ti}-\sum_{m=1}^{M} {\hat{\mathcal{B}}}_{tm}c_{im}-\sum_{s=1}^{S} {\hat{\mathcal{D}}}_{s}x_{is}-\sum_{q=1}^{Q} {\hat{\mathcal{C}}}_{q}z_{tiq}$ be the rank-specific residual corresponding to the $\mathcal{M}$ term, where $\hat{B}_{i}$, $\hat{\Gamma}$, $\hat{\Theta}_{i}$, ${\hat{\mathcal{B}}}_{tm}$, ${\hat{\mathcal{D}}}_{s}$, and ${\hat{\mathcal{C}}}_{q}$ are taken from the most recently sampled instances of their respective tensor margins. The $j$th element for margin $\mu_{d\cdot,r}$ for $j\in\left[ 2,p_{d}-1 \right]$, denoted $\mu_{d\cdot,r,j}$, follows the conditional posterior

$$\pi\left( \mu_{d\cdot,r,j} | - \right)\mathcal{=N}\left( \frac{n_{d\cdot,r,j}^{\mu}\tau^{\mu}w_{d,r}^{\mu}+e^{-\alpha_{d,r}^{\mu}}\left( \mu_{d\cdot,r,j-1}+\mu_{d\cdot,r,j+1} \right)}{m_{d\cdot,r,j}^{\mu}\tau^{\mu}w_{d,r}^{\mu}+1+e^{-{2\alpha}_{d,r}^{\mu}}}, \frac{\tau^{\mu}w_{d,r}^{\mu}}{m_{d\cdot,r,j}^{\mu}\tau^{\mu}w_{d,r}^{\mu}+1+e^{-{2\alpha}_{d,r}^{\mu}}} \right)$$

where $L_{d\cdot,r}^{\mu}=\mu_{1\cdot,r}\circ\ldots\circ\mu_{d-1\cdot,r}\circ\mu_{d+1\cdot,r}\circ\ldots\circ\mu_{D\cdot,r}$, $m_{d\cdot,r,j}^{\mu}=\sum_{i=1}^{n} \sum_{t=1}^{T_{i}} \frac{1}{\sigma_{\epsilon,ti}^{2}}\sum_{v^{'}\in\mathcal{V}_{ti,dj}} \left( L_{d\cdot,r}^{\mu}\left( v^{'} \right) \right)^{2}$, and $n_{d\cdot,r,j}^{\mu}=\sum_{i=1}^{n} \sum_{t=1}^{T_{i}} \frac{1}{\sigma_{\epsilon,ti}^{2}}\sum_{v^{'}\in\mathcal{V}_{ti,dj}} \mathcal{Y}_{ti,r,dj}^{\mu}\left( v^{'} \right)L_{d\cdot,r}^{\mu}\left( v^{'} \right)$. For $j\in\{1,p_{d}\}$, the posterior distribution is similar to above, but with the term $\left( \mu_{d\cdot,r,j-1}+\mu_{d\cdot,r,j+1} \right)$ in the numerator of the posterior mean being replaced by $\mu_{d\cdot,r,j\pm1}$, and the term $e^{-{2\alpha}_{d,r}^{\mu}}$ in the denominator of the posterior mean and variance being replaced by 0.

**Step 2**: For a given dimension $d\in\left[ 1,D \right]$ and rank $r\in\left[ 1,R \right]$, the diagonal entries of the tensor margin covariance matrix $\mathbf{W}_{d,r}^{\mu}$, denoted as $w_{d,r}^{\mu}$, follow the generalized inverse Gaussian (gIG) posterior $\pi\left( w_{d,r}^{\mu} | - \right)=\mathrm{gIG}\left( 1-\frac{p_{d}}{2},\chi=\frac{c_{d,r}^{\mu}}{1-e^{-2\alpha_{d,r}^{\mu}}},\psi=\lambda_{d,r}^{\mu} \right)$, where

$c_{d,r}^{\mu}=\frac{1}{\tau^{\mu}}\left\{ \sum_{j=2}^{p_{d}-1} \left( 1+e^{-2\alpha_{d,r}^{\mu}} \right)\left\| \mu_{d\cdot,r,j} \right\|_{2}^{2}+\left\| \mu_{d\cdot,r,1} \right\|_{2}^{2}+\left\| \mu_{d\cdot,r,p_{d}} \right\|_{2}^{2}-2e^{-\alpha_{d,r}^{\mu}}\sum_{j=1}^{p_{d}-1} {\mu_{d\cdot,r,j}}^{T}\mu_{d\cdot,r,j+1} \right\}$.

**Step 3**: The rate parameter $\lambda_{d,r}^{\mu}$ follows the conditional posterior $\pi\left( \lambda_{d,r}^{\mu} | - \right)=\mathrm{Ga}\left( a_{\lambda}+p_{d},b_{\lambda}+\frac{p_{d}w_{d,r}^{\mu}}{2} \right)$.

**Step 4**: The global variance scale parameter $\tau^{\mu}$ follows the conditional posterior

$$\pi\left( \tau^{\mu} | - \right)=\mathrm{gIG}\left( \mu=a_{\tau}-\frac{R\left( p_{1}+\ldots+p_{D} \right)}{2},\chi=\sum_{r=1}^{R} \sum_{d=1}^{D} \mu_{d\cdot,r}^{T}\left( \mathbf{W}_{d,r}^{\mu} \right)^{-1}\mu_{d\cdot,r},\psi=2b_{\tau} \right)$$

**Step 5**: The conditional posterior for lengthscale parameter $\alpha_{d,r}^{\mu}$ satisfies the following:

$$\pi\left( \alpha_{d,r}^{\mu} | - \right)\propto\left( \alpha_{d,r}^{\mu} \right)^{a_{\alpha}-1}\left( 1-e^{-2\alpha_{d,r}^{\mu}} \right)^{-\frac{1}{2}\left( p_{d}-1 \right)}\exp\left[ -\frac{1}{2}\left( \mu_{d\cdot,r}^{T}\left( \mathbf{W}_{d,r}^{\mu} \right)^{-1}\mu_{d\cdot,r}+2b_{\alpha}\alpha_{d,r}^{\mu} \right) \right]$$

Because this posterior does not correspond to a closed-form distribution, $\alpha_{d,r}^{\mu}$ is sampled using a Metropolis-Hastings (MH) step, using the proposal density $\alpha_{d,r,s_{x}+1}^{\mu}|\alpha_{d,r,s_{x}}^{\mu}\sim log-Normal(\alpha_{d,r,s_{x}}^{\mu},\sigma_{\alpha}^{2})$, where $s_{x}$ indexes the MCMC iteration, and $\sigma_{\alpha}^{2}$ is fixed for all $\alpha$ terms.

**Step 6**: Let $\mathcal{Y}_{ti,r}^{b}=\mathcal{Y}_{ti}-\hat{\mathcal{M}}-\hat{\Gamma}\times\mathcal{T}_{ti}-\hat{\Theta}_{i}\times\mathcal{T}_{ti}-\sum_{m=1}^{M} {\hat{\mathcal{B}}}_{tm}c_{im}-\sum_{s=1}^{S} {\hat{\mathcal{D}}}_{s}x_{is}-\sum_{q=1}^{Q} {\hat{\mathcal{C}}}_{q}z_{tiq}$

be the rank-specific residual corresponding to the $B_{i}$ term, where each tensor coefficient is taken from the most recently sampled instances of its respective tensor margins. For a given $i\in\left[ 1,n \right]$, the $j$th element for margin $b_{d\cdot,ri}$ for $j\in\left[ 2,p_{d}-1 \right]$ follows the conditional posterior

$$\pi\left( b_{d\cdot,ri,j} | - \right)\mathcal{=N}\left( \frac{n_{d\cdot,ri,j}^{b}{\tau_{i}^{b}w}_{d,r}^{b}+e^{-\alpha_{d,r}^{b}}\left( b_{d\cdot,ri,j-1}+b_{d\cdot,ri,j+1} \right)}{m_{d\cdot,ri,j}^{b}\tau_{i}^{b}w_{d,r}^{b}+1+e^{-{2\alpha}_{d,r}^{b}}}, \frac{\tau_{i}^{b}w_{d,r}^{b}}{m_{d\cdot,ri,j}^{b}\tau_{i}^{b}w_{d,r}^{b}+1+e^{-{2\alpha}_{d,r}^{b}}} \right)$$

where $L_{d\cdot,ri}^{b}=b_{1\cdot,ri}\circ\ldots\circ b_{d-1\cdot,ri}\circ b_{d+1\cdot,ri}\circ\ldots\circ b_{D\cdot,ri}$, $m_{d\cdot,ri,j}^{b}=\sum_{t=1}^{T_{i}} \frac{1}{\sigma_{\epsilon,ti}^{2}}\sum_{v^{'}\in\mathcal{V}_{ti,dj}} \left( L_{d\cdot,ri}^{b}\left( v^{'} \right) \right)^{2}$, and $n_{d\cdot,ri,j}^{b}=\sum_{t=1}^{T_{i}} \frac{1}{\sigma_{\epsilon,ti}^{2}}\sum_{v^{'}\in\mathcal{V}_{ti,dj}} \mathcal{Y}_{ti,r,dj}^{b}\left( v^{'} \right)L_{d\cdot,ri}^{b}\left( v^{'} \right)$. For $j\in\{1,p_{d}\}$, the posterior distribution is similar to above, but with the term $\left( b_{d\cdot,ri,j-1}+b_{d\cdot,ri,j+1} \right)$ in the numerator of the posterior mean being replaced by $b_{d\cdot,ri,j\pm1}$, and the term $e^{-{2\alpha}_{d,r}^{b}}$ in the denominator of the posterior mean and variance being replaced by 0.

**Step 7**: The diagonal entries of the tensor margin covariance matrix $\mathbf{W}_{d,r}^{b}$ follow the conditional posterior $\pi\left( w_{d,r}^{b} | - \right)=\mathrm{gIG}\left( 1-\frac{p_{d} n}{2},\chi=\frac{c_{d,r}^{b}}{1-e^{-2\alpha_{d,r}^{b}}},\psi=\lambda_{d,r}^{b} \right)$, where

$c_{d,r}^{b}=\sum_{i=1}^{n} \frac{1}{\tau_{i}^{b}}\left\{ \sum_{j=2}^{p_{d}-1} \left( 1+e^{-2\alpha_{d,r}^{b}} \right)\left\| b_{d\cdot,ri,j} \right\|_{2}^{2}+\left\| b_{d\cdot,ri,1} \right\|_{2}^{2}+\left\| b_{d\cdot,ri,p_{d}} \right\|_{2}^{2}-2e^{-\alpha_{d,r}^{b}}\sum_{j=1}^{p_{d}-1} {b_{d\cdot,ri,j}}^{T}b_{d\cdot,ri,j+1} \right\}$.

**Step 8**: The rate parameter $\lambda_{d,r}^{b}$ follows the conditional posterior $\pi\left( \lambda_{d,r}^{b} | - \right)=\mathrm{Ga}\left( a_{\lambda}+p_{d},b_{\lambda}+\frac{p_{d}w_{d,r}^{b}}{2} \right)$.

**Step 9**: The global variance scale parameter $\tau_{i}^{b}$ follows the conditional posterior

$$\pi\left( \tau_{i}^{b} | - \right)=\mathrm{gIG}\left( \mu=a_{\tau}-\frac{R\left( p_{1}+\ldots+p_{D} \right)}{2},\chi=\sum_{r=1}^{R} \sum_{d=1}^{D} b_{d\cdot,ri}^{T}\left( \mathbf{W}_{d,r}^{b} \right)^{-1}b_{d\cdot,ri},\psi=2b_{\tau} \right)$$

**Step 10**: The conditional posterior for parameter $\alpha_{d,r}^{b}$ satisfies the following:

$$\pi\left( \alpha_{d,r}^{b} | - \right)\propto\left( \alpha_{d,r}^{b} \right)^{a_{\alpha}-1}\left( 1-e^{-2\alpha_{d,r}^{b}} \right)^{-\frac{1}{2}\left( p_{d}-1 \right)n}\exp\left[ -\frac{1}{2}\left( \sum_{i=1}^{n} b_{d\cdot,ri}^{T}\left( \mathbf{W}_{d,r}^{b} \right)^{-1}b_{d\cdot,ri}+2b_{\alpha}\alpha_{d,r}^{b} \right) \right]$$

A MH step is used to sample $\alpha_{d,r}^{b}$ as in Step 5.

**Step 11**: Let $\mathcal{Y}_{ti,r}^{\gamma}=\mathcal{Y}_{ti}-\hat{\mathcal{M}}-\hat{B}_{i}-\hat{\Theta}_{i}\times\mathcal{T}_{ti}-\sum_{m=1}^{M} {\hat{\mathcal{B}}}_{tm}c_{im}-\sum_{s=1}^{S} {\hat{\mathcal{D}}}_{s}x_{is}-\sum_{q=1}^{Q} {\hat{\mathcal{C}}}_{q}z_{tiq}$ be the rank-specific residual corresponding to the $\Gamma$ term. The $j$th element for margin $\gamma_{d\cdot,r}$ for $j\in\left[ 2,p_{d}-1 \right]$ follows the conditional posterior

$$\pi\left( \gamma_{d\cdot,r,j} | - \right)\mathcal{=N}\left( \frac{n_{d\cdot,r,j}^{\gamma}\tau^{\gamma}w_{d,r}^{\gamma}+e^{-\alpha_{d,r}^{\gamma}}\left( \gamma_{d\cdot,r,j-1}+\gamma_{d\cdot,r,j+1} \right)}{m_{d\cdot,r,j}^{\gamma}\tau^{\gamma}w_{d,r}^{\gamma}+1+e^{-{2\alpha}_{d,r}^{\gamma}}}, \frac{\tau^{\gamma}w_{d,r}^{\gamma}}{m_{d\cdot,r,j}^{\gamma}\tau^{\gamma}w_{d,r}^{\gamma}+1+e^{-{2\alpha}_{d,r}^{\gamma}}} \right)$$

where $L_{d\cdot,r}^{\gamma}=\gamma_{1\cdot,r}\circ\ldots\circ\gamma_{d-1\cdot,r}\circ\gamma_{d+1\cdot,r}\circ\ldots\circ\gamma_{D\cdot,r}$, $m_{d\cdot,r,j}^{\gamma}=\sum_{i=1}^{n} \sum_{t=1}^{T_{i}} \frac{{\mathcal{T}_{ti}}^{2}}{\sigma_{\epsilon,ti}^{2}}\sum_{v^{'}\in\mathcal{V}_{ti,dj}} \left( L_{d\cdot,r}^{\gamma}\left( v^{'} \right) \right)^{2}$, and $n_{d\cdot,r,j}^{\gamma}=\sum_{i=1}^{n} \sum_{t=1}^{T_{i}} \frac{\mathcal{T}_{ti}}{\sigma_{\epsilon,ti}^{2}}\sum_{v^{'}\in\mathcal{V}_{ti,dj}} \mathcal{Y}_{ti,r,dj}^{\gamma}\left( v^{'} \right)L_{d\cdot,r}^{\gamma}\left( v^{'} \right)$. For $j\in\{1,p_{d}\}$, the posterior distribution is similar to above, but with the term $\left( \gamma_{d\cdot,r,j-1}+\gamma_{d\cdot,r,j+1} \right)$ in the numerator of the posterior mean being replaced by $\gamma_{d\cdot,r,j\pm1}$, and the term $e^{-{2\alpha}_{d,r}^{\gamma}}$ in the denominator of the posterior mean and variance being replaced by 0.

**Step 12**: The diagonal entries of the tensor margin covariance matrix $\mathbf{W}_{d,r}^{\gamma}$ follow the conditional posterior $\pi\left( w_{d,r}^{\gamma} | - \right)=\mathrm{gIG}\left( 1-\frac{p_{d}}{2},\chi=\frac{c_{d,r}^{\gamma}}{1-e^{-2\alpha_{d,r}^{\gamma}}},\psi=\lambda_{d,r}^{\gamma} \right)$, where

$c_{d,r}^{\gamma}=\frac{1}{\tau^{\gamma}}\left\{ \sum_{j=2}^{p_{d}-1} \left( 1+e^{-2\alpha_{d,r}^{\gamma}} \right)\left\| \gamma_{d\cdot,r,j} \right\|_{2}^{2}+\left\| \gamma_{d\cdot,r,1} \right\|_{2}^{2}+\left\| \gamma_{d\cdot,r,p_{d}} \right\|_{2}^{2}-2e^{-\alpha_{d,r}^{\gamma}}\sum_{j=1}^{p_{d}-1} {\gamma_{d\cdot,r,j}}^{T}\gamma_{d\cdot,r,j+1} \right\}$.

**Step 13**: The parameter $\lambda_{d,r}^{\gamma}$ follows the conditional posterior $\pi\left( \lambda_{d,r}^{\gamma} | - \right)=\mathrm{Ga}\left( a_{\lambda}+p_{d},b_{\lambda}+\frac{p_{d}w_{d,r}^{\gamma}}{2} \right)$.

**Step 14**: The global variance scale parameter $\tau^{\gamma}$ follows the conditional posterior

$$\pi\left( \tau^{\gamma} | - \right)=\mathrm{gIG}\left( \mu=a_{\tau}-\frac{R\left( p_{1}+\ldots+p_{D} \right)}{2},\chi=\sum_{r=1}^{R} \sum_{d=1}^{D} \gamma_{d\cdot,r}^{T}\left( \mathbf{W}_{d,r}^{\gamma} \right)^{-1}\gamma_{d\cdot,r},\psi=2b_{\tau} \right)$$

**Step 15**: The conditional posterior for parameter $\alpha_{d,r}^{\gamma}$ satisfies the following:

$$\pi\left( \alpha_{d,r}^{\gamma} | - \right)\propto\left( \alpha_{d,r}^{\gamma} \right)^{a_{\alpha}-1}\left( 1-e^{-2\alpha_{d,r}^{\gamma}} \right)^{-\frac{1}{2}\left( p_{d}-1 \right)}\exp\left[ -\frac{1}{2}\left( \gamma_{d\cdot,r}^{T}\left( \mathbf{W}_{d,r}^{\gamma} \right)^{-1}\gamma_{d\cdot,r}+2b_{\alpha}\alpha_{d,r}^{\gamma} \right) \right]$$

A MH step is used to sample $\alpha_{d,r}^{\gamma}$ as in Step 5.

**Step 16**: Let $\mathcal{Y}_{ti,r}^{\theta}=\mathcal{Y}_{ti}-\hat{\mathcal{M}}-\hat{B}_{i}-\hat{\Gamma}\times\mathcal{T}_{ti}-\sum_{m=1}^{M} {\hat{\mathcal{B}}}_{tm}c_{im}-\sum_{s=1}^{S} {\hat{\mathcal{D}}}_{s}x_{is}-\sum_{q=1}^{Q} {\hat{\mathcal{C}}}_{q}z_{tiq}$

be the rank-specific residual corresponding to the $\Theta_{i}$ term, where each tensor coefficient is taken from the most recently sampled instances of its respective tensor margins. For a given $i\in\left[ 1,n \right]$, the $j$th element for margin $\theta_{d\cdot,ri}$ for $j\in\left[ 2,p_{d}-1 \right]$ follows the conditional posterior

$$\pi\left( \theta_{d\cdot,ri,j} | - \right)\mathcal{=N}\left( \frac{n_{d\cdot,ri,j}^{\theta}{\tau_{i}^{\theta}w}_{d,r}^{\theta}+e^{-\alpha_{d,r}^{\theta}}\left( \theta_{d\cdot,ri,j-1}+\theta_{d\cdot,ri,j+1} \right)}{m_{d\cdot,ri,j}^{\theta}\tau_{i}^{\theta}w_{d,r}^{\theta}+1+e^{-{2\alpha}_{d,r}^{\theta}}}, \frac{\tau_{i}^{\theta}w_{d,r}^{\theta}}{m_{d\cdot,ri,j}^{\theta}\tau_{i}^{\theta}w_{d,r}^{\theta}+1+e^{-2\alpha_{d,r}^{\theta}}} \right)$$

where $L_{d\cdot,ri}^{\theta}=\theta_{1\cdot,ri}\circ\ldots\circ\theta_{d-1\cdot,ri}\circ\theta_{d+1\cdot,ri}\circ\ldots\circ\theta_{D\cdot,ri}$, $m_{d\cdot,ri,j}^{\theta}=\sum_{t=1}^{T_{i}} \frac{{\mathcal{T}_{ti}}^{2}}{\sigma_{\epsilon,ti}^{2}}\sum_{v^{'}\in\mathcal{V}_{ti,dj}} \left( L_{d\cdot,ri}^{\theta}\left( v^{'} \right) \right)^{2}$, and $n_{d\cdot,ri,j}^{\theta}=\sum_{t=1}^{T_{i}} \frac{\mathcal{T}_{ti}}{\sigma_{\epsilon,ti}^{2}}\sum_{v^{'}\in\mathcal{V}_{ti,dj}} \mathcal{Y}_{ti,r,dj}^{\theta}\left( v^{'} \right)L_{d\cdot,ri}^{\theta}\left( v^{'} \right)$. For $j\in\{1,p_{d}\}$, the posterior distribution is similar to above, but with the term $\left( \theta_{d\cdot,ri,j-1}+\theta_{d\cdot,ri,j+1} \right)$ in the numerator of the posterior mean being replaced by $\theta_{d\cdot,ri,j\pm1}$, and the term $e^{-{2\alpha}_{d,r}^{\theta}}$ in the denominator of the posterior mean and variance being replaced by 0.

**Step 17**: The diagonal entries of the tensor margin covariance matrix $\mathbf{W}_{d,r}^{\theta}$ follow the conditional posterior $\pi\left( w_{d,r}^{\theta} | - \right)=\mathrm{gIG}\left( 1-\frac{p_{d} n}{2},\chi=\frac{c_{d,r}^{\theta}}{1-e^{-2\alpha_{d,r}^{\theta}}},\psi=\lambda_{d,r}^{\theta} \right)$, where

$c_{d,r}^{\theta}=\sum_{i=1}^{n} \frac{1}{\tau_{i}^{\theta}}\left\{ \sum_{j=2}^{p_{d}-1} \left( 1+e^{-2\alpha_{d,r}^{\theta}} \right)\left\| \theta_{d\cdot,ri,j} \right\|_{2}^{2}+\left\| \theta_{d\cdot,ri,1} \right\|_{2}^{2}+\left\| \theta_{d\cdot,ri,p_{d}} \right\|_{2}^{2}-2e^{-\alpha_{d,r}^{\theta}}\sum_{j=1}^{p_{d}-1} {\theta_{d\cdot,ri,j}}^{T}\theta_{d\cdot,ri,j+1} \right\}$.

**Step 18**: The parameter $\lambda_{d,r}^{\theta}$ follows the conditional posterior $\pi\left( \lambda_{d,r}^{\theta} | - \right)=\mathrm{Ga}\left( a_{\lambda}+p_{d},b_{\lambda}+\frac{p_{d}w_{d,r}^{\theta}}{2} \right)$.

**Step 19**: The global variance scale parameter $\tau_{i}^{\theta}$ follows the conditional posterior

$$\pi\left( \tau_{i}^{\theta} | - \right)=\mathrm{gIG}\left( \mu=a_{\tau}-\frac{R\left( p_{1}+\ldots+p_{D} \right)}{2},\chi=\sum_{r=1}^{R} \sum_{d=1}^{D} \theta_{d\cdot,ri}^{T}\left( \mathbf{W}_{d,r}^{\theta} \right)^{-1}\theta_{d\cdot,ri},\psi=2b_{\tau} \right)$$

**Step 20**: The conditional posterior for parameter $\alpha_{d,r}^{\theta}$ satisfies the following:

$$\pi\left( \alpha_{d,r}^{\theta} | - \right)\propto\left( \alpha_{d,r}^{\theta} \right)^{a_{\alpha}-1}\left( 1-e^{-2\alpha_{d,r}^{\theta}} \right)^{-\frac{1}{2}\left( p_{d}-1 \right)n}\exp\left[ -\frac{1}{2}\left( \sum_{i=1}^{n} \theta_{d\cdot,ri}^{T}\left( \mathbf{W}_{d,r}^{\theta} \right)^{-1}\theta_{d\cdot,ri}+2b_{\alpha}\alpha_{d,r}^{\theta} \right) \right]$$

A MH step is used to sample $\alpha_{d,r}^{\theta}$ as in Step 5.

**Step 21**: Let $T=\max_{i=1}^{n} T_{i}$ be the maximum number of visits across subjects. For a given $m\in\left[ 1,M \right]$, let $\mathcal{Y}_{ti,rtm}^{\beta}=\mathcal{Y}_{ti}-\hat{\mathcal{M}}-\hat{B}_{i}-\hat{\Gamma}\times\mathcal{T}_{ti}-\hat{\Theta}_{i}\times\mathcal{T}_{ti}-\sum_{m^{*}=1,m^{*}\neq m}^{M} {\hat{\mathcal{B}}}_{tm^{*}}c_{im^{*}}-\sum_{s=1}^{S} {\hat{\mathcal{D}}}_{s}x_{is}-\sum_{q=1}^{Q} {\hat{\mathcal{C}}}_{q}z_{tiq}$ be the rank-specific residual corresponding to the $\mathcal{B}_{tm}$ term. Let $I_{t}$ be the set of subjects with a visit $t$, for $t\in\left[ 1,T \right]$. The $j$th element for margin $\beta_{d\cdot,rtm}$ for $j\in\left[ 2,p_{d}-1 \right]$ follows the conditional posterior

$$\pi\left( \beta_{d\cdot,rtm,j} | - \right)\mathcal{=N}\left( \frac{n_{d\cdot,rtm,j}^{\beta}{\tau_{tm}^{\beta}w}_{d,r}^{\beta}+e^{-\alpha_{d,r}^{\beta}}\left( \beta_{d\cdot,rtm,j-1}+\beta_{d\cdot,rtm,j+1} \right)}{m_{d\cdot,rtm,j}^{\beta}\tau_{tm}^{\beta}w_{d,r}^{\beta}+1+e^{-2\alpha_{d,r}^{\beta}}}, \frac{{\tau_{tm}^{\beta}w}_{d,r}^{\beta}}{m_{d\cdot,rtm,j}^{\beta}\tau_{tm}^{\beta}w_{d,r}^{\beta}+1+e^{-{2\alpha}_{d,r}^{\beta}}} \right)$$

where $L_{d\cdot,rtm}^{\beta}=\beta_{1\cdot,rtm}\circ\ldots\circ\beta_{d-1\cdot,rtm}\circ\beta_{d+1\cdot,rtm}\circ\ldots\circ\beta_{D\cdot,rtm}$, $m_{d\cdot,rtm,j}^{\beta}=\sum_{i\in I_{t}} \frac{c_{im}^{2}}{\sigma_{\epsilon,ti}^{2}}\sum_{v^{'}\in\mathcal{V}_{ti,dj}} \left( L_{d\cdot,ri}^{\beta}\left( v^{'} \right) \right)^{2}$, and $n_{d\cdot,rtm,j}^{\beta}=\sum_{i\in I_{t}} \frac{c_{im}}{\sigma_{\epsilon,ti}^{2}}\sum_{v^{'}\in\mathcal{V}_{ti,dj}} \mathcal{Y}_{ti,rtm,dj}^{\beta}\left( v^{'} \right)L_{d\cdot,rtm}^{\beta}\left( v^{'} \right)$. For $j\in\{1,p_{d}\}$, the posterior distribution is similar to above, but with the term $\left( \beta_{d\cdot,rtm,j-1}+\beta_{d\cdot,rtm,j+1} \right)$ in the numerator of the posterior mean being replaced by $\beta_{d\cdot,rtm,j\pm1}$, and the term $e^{-{2\alpha}_{d,r}^{\beta}}$ in the denominator of the posterior mean and variance being replaced by 0.

**Step 22**: The diagonal entries of the tensor margin covariance matrix $\mathbf{W}_{d,r}^{\beta}$ follow the conditional posterior $\pi\left( w_{d,r}^{\beta} | - \right)=\mathrm{gIG}\left( 1-\frac{p_{d}}{2},\chi=\frac{c_{d,r}^{\beta}}{1-e^{-2\alpha_{d,r}^{\beta}}},\psi=\lambda_{d,r}^{\beta} \right)$, where $c_{d,r}^{\beta}=\sum_{t=1}^{T} \sum_{m=1}^{M} c_{d,r,tm}^{\beta}$, with

$c_{d,r}^{\mu}=\frac{1}{\tau_{tm}^{\beta}}\left\{ \sum_{j=2}^{p_{d}-1} \left( 1+e^{-2\alpha_{d,r}^{\beta}} \right)\left\| \beta_{d\cdot,rtm,j} \right\|_{2}^{2}+\left\| \beta_{d\cdot,rtm,1} \right\|_{2}^{2}+\left\| \beta_{d\cdot,rtm,p_{d}} \right\|_{2}^{2}-2e^{-\alpha_{d,r}^{\beta}}\sum_{j=1}^{p_{d}-1} {\beta_{d\cdot,rtm,j}}^{T}\beta_{d\cdot,rtm,j+1} \right\}$.

**Step 23**: The parameter $\lambda_{d,r}^{\beta}$ follows the conditional posterior $\pi\left( \lambda_{d,r}^{\beta} | - \right)=\mathrm{Ga}\left( a_{\lambda}+p_{d},b_{\lambda}+\frac{p_{d}w_{d,r}^{\beta}}{2} \right)$.

**Step 24**: The parameter $\tau_{tm}^{\beta}$ follows the conditional posterior

$$\pi\left( \tau_{tm}^{\beta} | - \right)=\mathrm{gIG}\left( \mu=a_{\tau}-\frac{R\left( p_{1}+\ldots+p_{D} \right)}{2},\chi=\sum_{r=1}^{R} \sum_{d=1}^{D} \beta_{d\cdot,rtm}^{T}\left( \mathbf{W}_{d,r}^{\beta} \right)^{-1}\beta_{d\cdot,rtm},\psi=2b_{\tau} \right)$$

**Step 25**: The conditional posterior for parameter $\alpha_{d,r}^{\beta}$ is proportional to the following expression:

$$\left( \alpha_{d,r}^{\beta} \right)^{a_{\alpha}-1}\left( 1-e^{-2\alpha_{d,r}^{\beta}} \right)^{-\frac{1}{2}TM\left( p_{d}-1 \right)}\exp\left[ -\frac{1}{2}\left( \sum_{t=1}^{T} \sum_{m=1}^{M} \beta_{d\cdot,rtm}^{T}\left( W_{d,r}^{\beta} \right)^{-1}\beta_{d\cdot,rtm}+2b_{\alpha}\alpha_{d,r}^{\beta} \right) \right]$$

A MH step is used to sample $\alpha_{d,r}^{\beta}$ as in Step 5.

**Step 26**: For a given $s\in\left[ 1,S \right]$, let $\mathcal{Y}_{ti,rs}^{\delta}=\mathcal{Y}_{ti}-\hat{\mathcal{M}}-\hat{B}_{i}-\hat{\Theta}_{i}\times\mathcal{T}_{ti}-\hat{\Gamma}\times\mathcal{T}_{ti}-\sum_{m=1}^{M} {\hat{\mathcal{B}}}_{tm}c_{im}-\sum_{s^{*}=1,s^{*}\neq s}^{S} {\hat{\mathcal{D}}}_{s^{*}}x_{is^{*}}-\sum_{q=1}^{Q} {\hat{\mathcal{C}}}_{q}z_{tiq}$ be the rank-specific residual corresponding to the $\mathcal{D}_{s}$ term. The $j$th element for margin $\delta_{d\cdot,rs}$ for $j\in\left[ 2,p_{d}-1 \right]$ follows the conditional posterior

$$\pi\left( \delta_{d\cdot,rs,j} | - \right)\mathcal{=N}\left( \frac{n_{d\cdot,rs,j}^{\delta}{\tau_{s}^{\delta}w}_{d,r}^{\delta}+e^{-\alpha_{d,r}^{\delta}}\left( \delta_{d\cdot,rs,j-1}+\delta_{d\cdot,rs,j+1} \right)}{m_{d\cdot,rs,j}^{\delta}\tau_{s}^{\delta}w_{d,r}^{\delta}+1+e^{-{2\alpha}_{d,r}^{\delta}}}, \frac{{\tau_{s}^{\delta}w}_{d,r}^{\delta}}{m_{d\cdot,rs,j}^{\delta}\tau_{s}^{\delta}w_{d,r}^{\delta}+1+e^{-{2\alpha}_{d,r}^{\delta}}} \right)$$

where $L_{d\cdot,rs}^{\delta}=\delta_{1\cdot,rs}\circ\ldots\circ\delta_{d-1\cdot,rs}\circ\delta_{d+1\cdot,rs}\circ\ldots\circ\delta_{D\cdot,rs}$, $m_{d\cdot,rs,j}^{\delta}=\sum_{i=1}^{n} \frac{{x_{is}}^{2}}{\sigma_{\epsilon,ti}^{2}}\sum_{v^{'}\in\mathcal{V}_{ti,dj}} \left( L_{d\cdot,rs}^{\delta}\left( v^{'} \right) \right)^{2}$, and $n_{d\cdot,rs,j}^{\delta}=\sum_{i=1}^{n} \frac{x_{is}}{\sigma_{\epsilon,ti}^{2}}\sum_{v^{'}\in\mathcal{V}_{ti,dj}} \mathcal{Y}_{ti,rs,dj}^{\delta}\left( v^{'} \right)L_{d\cdot,rs}^{\delta}\left( v^{'} \right)$. For $j\in\{1,p_{d}\}$, the posterior distribution is similar to above, but with the term $\left( \delta_{d\cdot,rs,j-1}+\delta_{d\cdot,rs,j+1} \right)$ in the numerator of the posterior mean being replaced by $\delta_{d\cdot,rs,j\pm1}$, and the term $e^{-{2\alpha}_{d,r}^{\delta}}$ in the denominator of the posterior mean and variance being replaced by 0.

**Step 27**: The diagonal entries of the tensor margin covariance matrix $\mathbf{W}_{d,r}^{\delta}$ follow the conditional posterior $\pi\left( w_{d,r}^{\delta} | - \right)=\mathrm{gIG}\left( 1-\frac{p_{d}}{2},\chi=\frac{c_{d,r}^{\delta}}{1-e^{-2\alpha_{d,r}^{\delta}}},\psi=\lambda_{d,r}^{\delta} \right)$, where

$c_{d,r}^{\delta}=\sum_{s=1}^{S} \frac{1}{\tau_{s}^{\delta}}\left\{ \sum_{j=2}^{p_{d}-1} \left( 1+e^{-2\alpha_{d,r}^{\delta}} \right)\left\| \delta_{d\cdot,rs,j} \right\|_{2}^{2}+\left\| \delta_{d\cdot,rs,1} \right\|_{2}^{2}+\left\| \delta_{d\cdot,rs,p_{d}} \right\|_{2}^{2}-2e^{-\alpha_{d,r}^{\delta}}\sum_{j=1}^{p_{d}-1} {\delta_{d\cdot,rs,j}}^{T}\delta_{d\cdot,rs,j+1} \right\}$.

**Step 28**: The parameter $\lambda_{d,r}^{\delta}$ follows the conditional posterior $\pi\left( \lambda_{d,r}^{\delta} | - \right)=\mathrm{Ga}\left( a_{\lambda}+p_{d},b_{\lambda}+\frac{p_{d}w_{d,r}^{\delta}}{2} \right)$.

**Step 29**: The global variance scale parameter $\tau_{s}^{\delta}$ follows the conditional posterior

$$\pi\left( \tau_{s}^{\delta} | - \right)=\mathrm{gIG}\left( \mu=a_{\tau}-\frac{R\left( p_{1}+\ldots+p_{D} \right)}{2},\chi=\sum_{r=1}^{R} \sum_{d=1}^{D} \delta_{d\cdot,rs}^{T}\left( \mathbf{W}_{d,r}^{\delta} \right)^{-1}\delta_{d\cdot,rs},\psi=2b_{\tau} \right)$$

**Step 30**: The conditional posterior for parameter $\alpha_{d,r}^{\delta}$ satisfies the following:

$$\pi\left( \alpha_{d,r}^{\delta} | - \right)\propto\left( \alpha_{d,r}^{\delta} \right)^{a_{\alpha}-1}\left( 1-e^{-2\alpha_{d,r}^{\delta}} \right)^{-\frac{1}{2} S \left( p_{d}-1 \right)}\exp\left[ -\frac{1}{2}\left( \sum_{s=1}^{S} \delta_{d\cdot,rs}^{T}\left( \mathbf{W}_{d,r}^{\delta} \right)^{-1}\delta_{d\cdot,rs}+2b_{\alpha}\alpha_{d,r}^{\delta} \right) \right]$$

A MH step is used to sample $\alpha_{d,r}^{\delta}$ as in Step 5.

**Step 31**: $\mathcal{C}_{q}$ and the associated parameters are sampled similar as the steps related to time-slope term $\Gamma$, looping through covariates $z_{tiq},q=1,\ldots,Q$.

**Step 32**: Lastly, the residual variance term $\sigma_{\epsilon,ti}^{2}$ is sampled as follows. Let $\mathcal{R}_{ti}=\mathcal{Y}_{ti}-\hat{\mathcal{M}}-\hat{B}_{i}-\hat{\Theta}_{i}\times\mathcal{T}_{ti}-\hat{\Gamma}\times\mathcal{T}_{ti}-\sum_{m=1}^{M} {\hat{\mathcal{B}}}_{tm}c_{im}-\sum_{s=1}^{S} {\hat{\mathcal{D}}}_{s}x_{is}-\sum_{q=1}^{Q} {\hat{\mathcal{C}}}_{q}z_{tiq}$ be the residual at the current iteration, denote $\mathcal{V}_{i}$ as the set of observed voxels for the $i$th subject, and denote $T_{i}$ as the number of visits for the $i$th subject. Then the conditional posterior for $\sigma_{\epsilon,ti}^{2}$ for $i\in\left[ 1,n \right]$ and $t\in\left[ 1,T_{i} \right]$ is given by

$$\pi(\sigma_{\epsilon,ti}^{2}|-) = Inverse-Gamma \left( a_{\epsilon}+\frac{\left| \mathcal{V}_{ti} \right|}{2},b_{\epsilon}+\frac{1}{2}\sum_{v\in\mathcal{V}_{i}} \mathcal{R}_{ti}^{2}\left( v \right) \right)$$

**Cluster Extent Inference**

For both the simulated and real datasets, we utilize Cluster-Extent Inference for multiplicity adjustments after fitting the voxel-wise regression models. The particular implementation we chose works as follows. Given a statistical parametric map (e.g. 3D map of t-statistics), the method first identifies connected regions (i.e. clusters) of voxels with values above some pre-specified threshold, where each cluster can be described by its height (i.e. max value) and its spatial extent (i.e. number of voxels). The iterative Benjamini-Hochberg procedure is used to select optimal thresholding choices corresponding to the height and spatial extent such that the false discovery rate (FDR) – i.e. the proportion of expected number of false-positive clusters to all regions declared positive – falls below a specified value, on average (Chumbley et al., 2010; Chumbley and Friston, 2009; Benjamini and Hochberg, 1995). After doing so, adjusted p-values can be obtained at the cluster-level. The rftResults function within the ANTsR package was used to implement this CEI approach (Tustison et al., 2021), with an initial threshold (Type I error rate) and cluster FDR set to $\alpha$ = 0.05.

**Additional Simulation Results**

Here, we present various details regarding the simulation studies that could not be contained in the main manuscript. Figure S1 shows an example of the DIC score versus fitted rank for a Scheme 1 replicate, with true coefficients generated from a rank-2 PARAFAC decomposition. We note that the optimal rank was chosen by finding the fitted rank with the lowest DIC score, which in this case was rank 4. While this choice of rank did not recover the true rank used to generate model coefficients, it led to better out-of-sample prediction and feature selection than fitted ranks of 1 or 2 using the l-BTRR method.

Table S1 displays the coefficient accuracy (c-RMSE) and feature selection metrics based on a thin strip along the boundary of the Scheme 2 signals, with reported results averaged across 50 replicates. We note that for any given method, both coefficient accuracy and feature selection are worse along this thin strip than they are for the full signals (see Table 4 in the main manuscript for comparison. However, the improved estimation accuracy and feature selection of the tensor-based l-BTRR method over the voxel-wise alternatives (after multiplicity correction) is still present when examining signals along their discontinuous boundaries. This indicates that the proposed method is preferable for respecting the sharp discontinuities in true signals compared with the voxel-wise approaches.

In Tables S2-S4, the feature selection, coverage probabilities, and coverage interval widths are reported for Schemes 1-3, but without applying multiplicity adjustments on the significance estimates of each method. In particular, the significance estimates for the l-BTRR and cs-BTRR methods are obtained by finding the 95% pointwise credible intervals for each voxel of the fitted model coefficients and outcome, and vl-OLS and vcs-OLS estimates come from the uncorrected p-values corresponding the t-statistics at each voxel of the fitted coefficients and outcome. We note that without applying multiplicity corrections, the voxel-wise approaches outperform the tensor-based approaches in terms of feature selection, but no method has higher F1 score compared with the multiplicity-adjusted l-BTRR results presented in the main manuscript (Tables 3-5).

**Additional Aphasia Results**

The posterior sampling algorithm also requires us to tune a variance parameter, $\sigma_{\alpha}^{2}$, which is used for the Metropolis-Hastings step when sampling lengthscale parameter $\alpha$ (see Step 5 in Posterior Computation). In order to do so, we explored the MCMC convergence for 3 different choices of $\sigma_{\alpha}^{2}$ (i.e. 0.01, 0.05, and 0.1), and chose the value that resulted in the best mixing, i.e. 0.01. Table S5 illustrates this finding by showing the effective sample size (ESS) for these choices of $\sigma_{\alpha}^{2}$ under the l-BTRR and cs-BTRR methods, fit to the Aphasia dataset using rank 3, 5000 MCMC iterations, and a burn-in rate of 0.5.

After assessing convergence and tuning parameters, we also examined correlations in the fitted AUC outcome across the spatially configured voxels using the l-BTRR method. In Figure S2, the distributions of pairwise voxel correlations are plotted as a function of distance (i.e. radius) between voxels, both for a tensor-valued model coefficient (panel a.) and the fitted AUC outcome (panel b.) under Model (6) in the main manuscript. As expected, the mean correlation for voxels that are spatially-distant is lower than that of voxels which are close in proximity – this is a consequence of both the low-rank decomposition assumed for each model coefficient and the prior structure which imposes exponentially-decreasing correlations across elements of each coefficient tensor margin. Note that the mean correlations between elements of the fitted AUC outcome (i.e. panel b. of Figure S2) decreases faster towards 0 as the radius increases, compared with the correlations among voxels of a single model coefficient (i.e. panel a. of Figure S2). This finding is also expected, given that lower-rank tensor objects, such as a single model coefficient, induce greater overall correlations between proximal voxels than higher-rank objects, such as the fitted AUC outcome. However, even though in both of these cases, mean correlation between voxels decreases as radius increases, the tails of the boxplots indicate that some relatively strong correlations exist even between spatially distant voxels. This can be explained by the fact that the sampled posteriors result from a combination of the priors in (2) and the likelihood functions stemming from the observed Aphasia dataset, the latter of which may have spatially distant voxels with high correlations. To that end, we also examined correlations in fitted AUC across the two brain hemispheres and found a substantial proportion of voxels exhibiting moderate to high correlations with their cross-hemisphere counterparts, even though these voxels were not necessarily spatially close (see Figure S3). Taken together, these findings illustrate the flexibility of the proposed l-BTRR method in terms of inducing spatial clustering while still preserving potentially relevant biological structure that does not relate to spatial proximity.

# **References**

Benjamini, Y., & Hochberg, Y. (1995). Controlling the false discovery rate: a practical and powerful approach to multiple testing. *Journal of the Royal Statistical Society: Series B (Methodological)*, **57**(1), 289-300.

Chumbley, J., Worsley, K, Flandin, G., & Friston, K. (2010). Topological FDR for neuroimaging. *Neuroimage*, **49**(4), 3057–3064.

Chumbley, J., & Friston, K. (2009). False discovery rate revisited: FDR and topological inference using Gaussian random fields. *Neuroimage*, **44**(1), 62-70.

Shriner, D., & Yi, N. (2009). Deviance information criterion (DIC) in Bayesian multiple QTL mapping. *Computational Statistics & Data Analysis*, **53**(5), 1850–1860.

Tustison, N., Cook, P., Holbrook, A., Johnson, H., Muschelli, J., Devenyi, G., Duda, J., Das, S., Cullen, N., Gillen, D., Yassa, M., Stone, J., Gee, J., & Avants, B. (2021). The ANTsX ecosystem for quantitative biological and medical imaging. *Scientific reports*, **11**(1), 1-13.

# **Supplementary Tables**

Table S1: Simulation results for estimation along the signal boundaries under Scheme 2. Coefficient estimation RMSE and feature selection metrics are shown.

| Sphere: Holdout 25% | | | | |
| --- | --- | --- | --- | --- |
| Method | c-RMSE | Sens | Spec | F1 |
| l-BTRR | **0.256** | 0.932 | 0.795 | **0.857** |
| cs-BTRR | 0.279 | 0.899 | 0.835 | 0.847 |
| vl-OLS | 0.367 | 0.252 | **0.999** | 0.384 |
| vcs-OLS | 0.366 | 0.258 | **0.999** | 0.392 |
| vl-Lasso | 0.368 | **0.977** | 0.151 | 0.380 |
| vcs-Lasso | 0.366 | 0.834 | 0.586 | 0.590 |
| Sphere: Holdout 50% | | | | |
| l-BTRR | **0.259** | 0.919 | 0.800 | **0.850** |
| cs-BTRR | 0.280 | 0.889 | 0.814 | 0.831 |
| vl-OLS | 0.386 | 0.226 | **0.999** | 0.353 |
| vcs-OLS | 0.386 | 0.239 | **0.999** | 0.369 |
| vl-Lasso | 0.388 | **0.958** | 0.153 | 0.377 |
| vcs-Lasso | 0.387 | 0.819 | 0.579 | 0.556 |
| Cube: Holdout 25% | | | | |
| l-BTRR | **0.120** | 0.941 | 0.845 | **0.865** |
| cs-BTRR | 0.127 | 0.845 | 0.926 | 0.825 |
| vl-OLS | 0.345 | 0.279 | **0.999** | 0.423 |
| vcs-OLS | 0.345 | 0.300 | **0.999** | 0.445 |
| vl-Lasso | 0.349 | **0.970** | 0.168 | 0.420 |
| vcs-Lasso | 0.344 | 0.846 | 0.557 | 0.539 |
| Cube: Holdout 50% | | | | |
| l-BTRR | **0.121** | 0.918 | 0.845 | **0.846** |
| cs-BTRR | 0.130 | 0.804 | 0.957 | 0.802 |
| vl-OLS | 0.364 | 0.253 | **0.999** | 0.390 |
| vcs-OLS | 0.363 | 0.271 | **0.999** | 0.411 |
| vl-Lasso | 0.367 | **0.961** | 0.161 | 0.385 |
| vcs-Lasso | 0.363 | 0.830 | 0.549 | 0.519 |

Table S2: **Scheme 1 results (no multiplicity)**. Feature selection (Sens, Spec, F1), and coverage probability for 4 competing methods. For feature selection, metrics are shown without multiplicity correction, where pointwise credible intervals are used for tensor-based approaches (l-BTRR and cs-BTRR), and uncorrected p-values are used for voxel-wise OLS approaches.

| Holdout 25% | | | | |
| --- | --- | --- | --- | --- |
| Method | Sens | Spec | F1 | Coverage |
| l-BTRR | 0.996 | 0.745 | 0.724 | 0.559 (0.470) |
| cs-BTRR | **1.000** | 0.503 | 0.577 | 0.230 (**0.341**) |
| vl-OLS | 0.904 | 0.809 | 0.825 | **0.795** (2.102) |
| vcs-OLS | 0.835 | **0.945** | **0.835** | 0.697 (1.758) |
| Holdout 50% | | | | |
| l-BTRR | 0.998 | 0.743 | 0.724 | 0.549 (0.481) |
| cs-BTRR | **1.000** | 0.494 | 0.573 | 0.223 (**0.328**) |
| vl-OLS | 0.770 | **0.949** | 0.800 | **0.795** (2.101) |
| vcs-OLS | 0.798 | 0.946 | **0.813** | 0.697 (1.756) |

Table S3: **Scheme 2 results (no multiplicity)**. Feature selection (Sens, Spec, F1), and coverage probability for 4 competing methods. For feature selection, metrics are shown without multiplicity correction, where pointwise credible intervals are used for tensor-based approaches (l-BTRR and cs-BTRR), and uncorrected p-values are used for voxel-wise OLS approaches.

| Sphere: Holdout 25% | | | | |
| --- | --- | --- | --- | --- |
| Method | Sens | Spec | F1 | Coverage |
| l-BTRR | 0.991 | 0.505 | 0.574 | 0.497 (0.545) |
| cs-BTRR | **0.999** | 0.406 | 0.532 | 0.302 (**0.286**) |
| vl-OLS | 0.797 | **0.952** | 0.819 | **0.798** (2.015) |
| vcs-OLS | 0.819 | 0.948 | **0.829** | 0.713 (1.744) |
| Sphere: Holdout 50% | | | | |
| l-BTRR | 0.992 | 0.495 | 0.569 | 0.470 (0.517) |
| cs-BTRR | **0.999** | 0.402 | 0.529 | 0.299 (**0.287**) |
| vl-OLS | 0.757 | **0.951** | 0.793 | **0.798** (2.014) |
| vcs-OLS | 0.781 | 0.949 | **0.806** | 0.713 (1.744) |
| Cube: Holdout 25% | | | | |
| l-BTRR | 0.978 | 0.805 | 0.787 | 0.668 (0.477) |
| cs-BTRR | **1.000** | 0.592 | 0.650 | 0.363 (**0.323**) |
| vl-OLS | 0.808 | **0.952** | 0.832 | **0.798** (2.090) |
| vcs-OLS | 0.826 | 0.948 | **0.838** | 0.710 (1.767) |
| Cube: Holdout 50% | | | | |
| l-BTRR | 0.998 | 0.780 | 0.781 | 0.666 (0.472) |
| cs-BTRR | **1.000** | 0.564 | 0.634 | 0.348 (**0.301**) |
| vl-OLS | 0.766 | **0.951** | 0.805 | **0.798** (2.087) |
| vcs-OLS | 0.786 | 0.948 | **0.814** | 0.710 (1.766) |

Table S4: **Scheme 3 results (no multiplicity)**. Feature selection (Sens, Spec, F1), and coverage probability for 4 competing methods. For feature selection, metrics are shown without multiplicity correction, where pointwise credible intervals are used for tensor-based approaches (l-BTRR and cs-BTRR), and uncorrected p-values are used for voxel-wise OLS approaches.

| Sphere: Holdout 25% | | | | |
| --- | --- | --- | --- | --- |
| Method | Sens | Spec | F1 | Coverage |
| l-BTRR | 0.938 | 0.589 | 0.531 | 0.514 (0.522) |
| cs-BTRR | **0.992** | 0.446 | 0.486 | 0.332 (**0.301**) |
| vl-OLS | 0.398 | 0.935 | 0.646 | **0.789** (2.095) |
| vcs-OLS | 0.444 | **0.937** | **0.780** | 0.710 (1.743) |
| Sphere: Holdout 50% | | | | |
| l-BTRR | 0.913 | 0.585 | 0.514 | 0.483 (0.457) |
| cs-BTRR | **0.995** | 0.437 | 0.482 | 0.329 (**0.303**) |
| vl-OLS | 0.372 | 0.920 | 0.596 | **0.789** (2.095) |
| vcs-OLS | 0.430 | **0.935** | **0.694** | 0.709 (1.743) |
| Cube: Holdout 25% | | | | |
| l-BTRR | 0.972 | 0.752 | 0.693 | 0.649 (0.452) |
| cs-BTRR | **0.997** | 0.524 | 0.555 | 0.358 (**0.309**) |
| vl-OLS | 0.390 | 0.932 | 0.640 | **0.789** (2.217) |
| vcs-OLS | 0.441 | **0.934** | **0.713** | 0.703 (1.754) |
| Cube: Holdout 50% | | | | |
| l-BTRR | 0.957 | 0.736 | 0.676 | 0.627 (0.466) |
| cs-BTRR | **0.998** | 0.501 | 0.542 | 0.345 (**0.302**) |
| vl-OLS | 0.360 | 0.914 | 0.587 | **0.788** (2.218) |
| vcs-OLS | 0.427 | **0.933** | **0.699** | 0.702 (1.755) |

Table S5: Effective Sample Size (ESS) for different choices of Metropolis Hastings proposal density width term, $\sigma_{\alpha}^{2}$. ESS are shown for l-BTRR and cs-BTRR methods out of 2500 post-burn-in MCMC samples for the Aphasia application. Mean and standard deviation of ESS are shown by looking across all observed voxels.

| Method | $\sigma_{\alpha}^{2}=0.01$ | $\sigma_{\alpha}^{2}=0.05$ | $\sigma_{\alpha}^{2}=0.10$ |
| --- | --- | --- | --- |
| l-BTRR | 1940.3 (452.2) | 1498.2 (453.8) | 1494.4 (304.8) |
| cs-BTRR | 2458 (290.7) | 2400.5 (422.2) | 2418.2 (436.9) |

# **Supplementary Figures**

Figure S1: Average Deviance Information Criteria (DIC) across 50 replicates from Scheme 1 for coefficient ranks of 1-5 fit using l-BTRR. True coefficients are generated from a PARAFAC decomposition with rank 2.


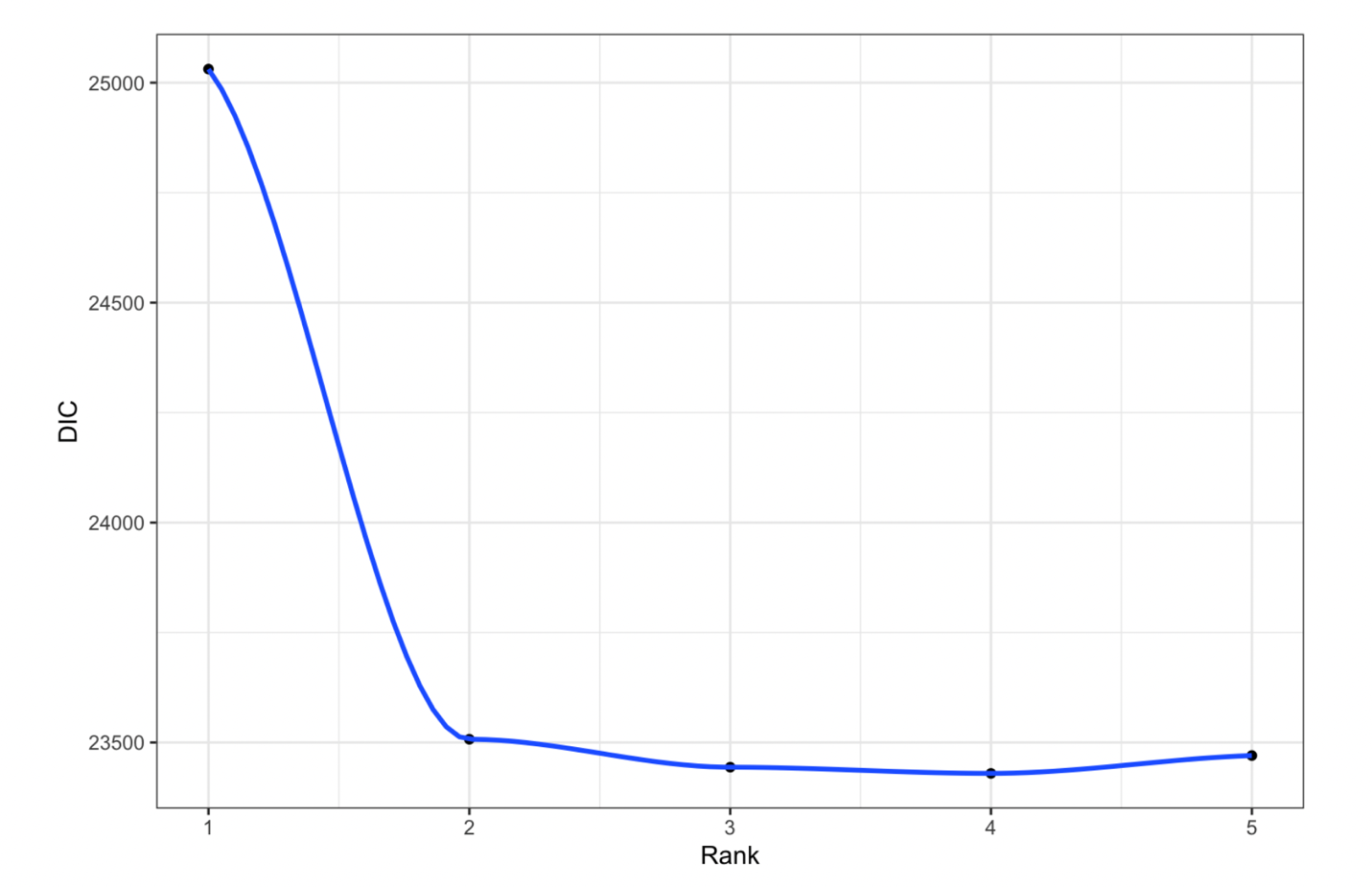


Figure S2: Spatial correlations by proximity in sampled coefficient $\mathcal{D}_{1}$ (panel a) and the sampled AUC outcome (panel b) for the Aphasia analysis. Boxplots were generated by finding the distribution of mean correlations between a center voxel and other voxels within a ring of given radius, which is repeated by treating each voxel in the brain mask as a center voxel.


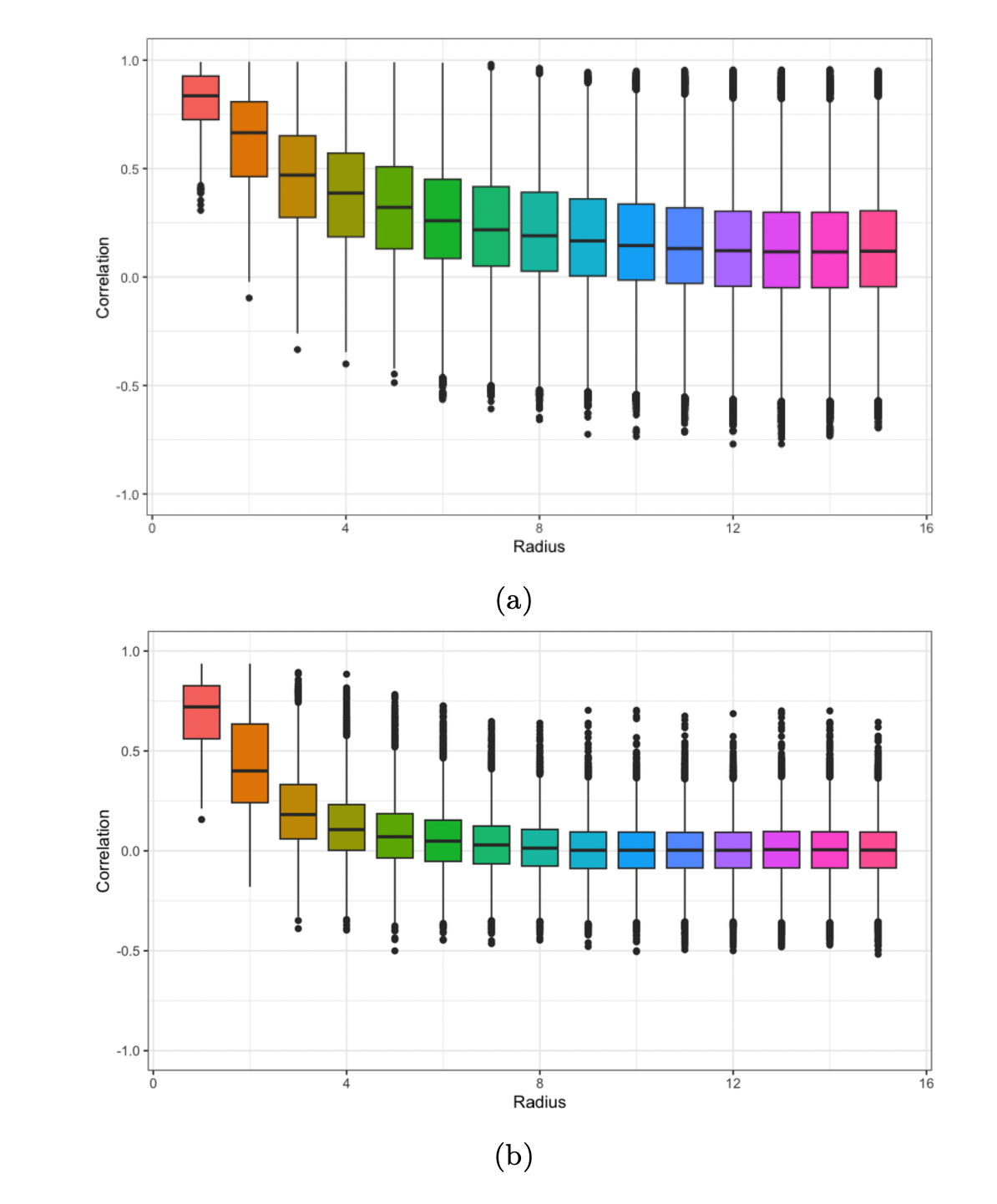


Figure S3: Histogram of cross-hemisphere correlations in fitted AUC outcome for Aphasia analysis, using MCMC samples post-burn-in to compute correlations between a given voxel and its cross-hemisphere counterpart.


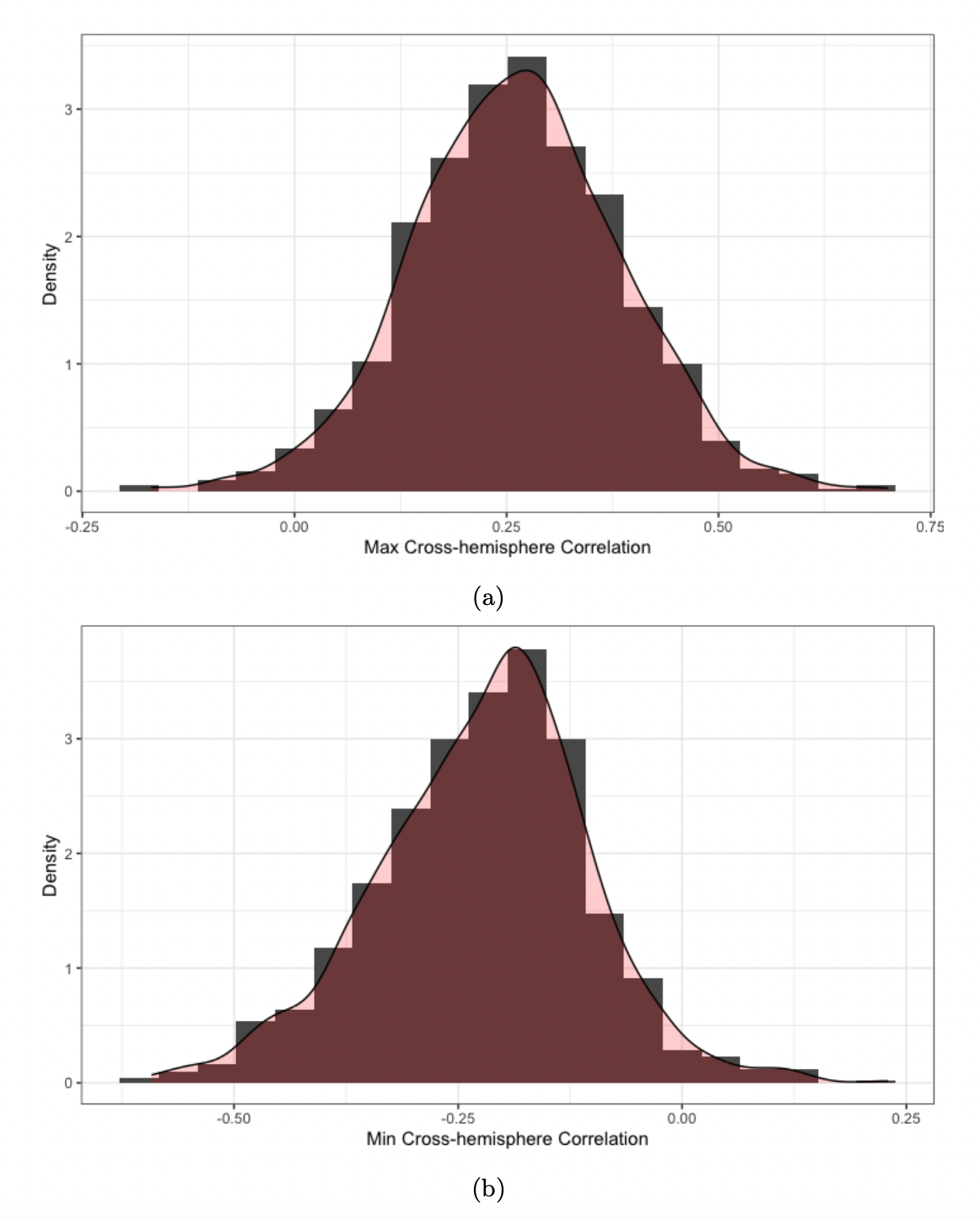


1. Corresponding author [↑](#footnote-ref-1)
